# Youth Perceptions of Male Homosexuality: Conceptions and Stereotypes in Brazil

**DOI:** 10.1007/s10508-025-03206-2

**Published:** 2025-08-05

**Authors:** Emerson Vicente-Cruz, Maria T. Soto-Sanfiel, Ariadna Angulo-Brunet

**Affiliations:** 1 Departament de Psicologia Social i Psicologia Quantitativa, Universitat de Barcelona, Barcelona, Spain; 2 Department of Communications & New Media, National University of Singapore, Singapore, Singapore; 3 Estudis de Psicologia i Ciències de l’Educació, Universitat Oberta de Catalunya, Campus UOC, Rambla de Poblenou, 156, 08018 Barcelona, Spain

**Keywords:** Adolescents, Stereotypes, Gays, Homophobia, Sexual orientation, Religion

## Abstract

This study identifies traditional conceptions of homosexuality and social stereotypes about gay men among Brazilian teenagers. A total of 178 adolescents (50% girls; age range 11–16 years) participated in 45 focus groups held at five schools in Brazil, in which a semi-structured script and videos depicting homophobic bullying were used to elicit their opinions. Thematic analysis revealed a dichotomy in their conceptions and stereotypes: While some spontaneously expressed homophobic conceptions (e.g., homosexuality is a sin or a pathology) supported by stereotypes (e.g., gays are not real men or sexually harass heterosexuals), others openly condemned homophobia and actively voiced their objections to the former group’s homophobic views. The analysis also reveals the persisting influence of religious beliefs in their perceptions. The study highlights the need to foster critical reflections in educational settings and the formulation of strategies to deconstruct homophobia and promote diversity and inclusion.

## Introduction

The impact of social stereotypes of gay males in the teenage education context is a crucial aspect of studies on homosexuality. Recent research has warned about the dangers of the normalization and trivialization of homophobia during adolescence, which can generate deeply ingrained preconceptions and stereotypes of male homosexuality (e.g., Baams & Kaufman, 2023; Elipe et al., 2022; Vicente-Cruz et al., 2025). Stereotypes encompass both positive and negative expectations about the characteristics and behaviors of certain social groups or their members (Morrison & Bearden, 2007), including sexually diverse communities, which affect the way information is processed (Heinze & Horn, 2014). This study identifies traditional conceptions about homosexuality and how these shape their stereotypes of gay men among Brazilian adolescents.

Brazil has the disheartening distinction of being the global leader in LGBTQ+ murders and homicides (Observatório de Mortes e Violências LGBTI+ no Brasil, 2024). A growing number of studies have explored the social traditional conceptions of male homosexuality in the country, specifically those influenced by religion (e.g., Barreiros & Brêtas, 2024; Sartori & Miranda, 2023) and beliefs that pathologize LGBTQ+ individuals (Vezzosi et al., 2019). Although previous research has addressed conceptions of male homosexuality in Brazil, none has specifically focused on stereotypical views of gay males among young Brazilians. This information would be useful for two main reasons. First, it would benefit the healthy formation of gender and sexual identities in such a crucial stage as adolescence. Second, it would support the formulation of education strategies that promote respect and inclusion regarding sexual and gender diversity.

### Traditional Conceptions of Male Homosexuality in Brazil

Conceptions of homosexuality have historically been interpreted in the light of prevailing paradigms, which in turn reflect social and cultural beliefs and their transformation. In this study, the term “traditional conceptions” refers to historical frameworks for understanding homosexuality, encompassing medical, religious and social discourses. While these conceptions may be influenced by gender norms, they extend beyond “hegemonic masculinity,” drawing from a variety of systems of knowledge and power.

Conventionally, the conceptualization of homosexuality has been used as a tool for control (Foucault, 1985), often linked to sin and immorality (Souza et al., 2017). This conception was subsequently reevaluated from the fields of biology and psychology, leading to debates about the innate versus constructed nature of homosexuality in contemporary society, along with efforts to destigmatize it and have it removed from diagnostic manuals. Lacerda et al. (2002) present more inclusive perspectives, arguing that homosexuality should not be considered a pathology. However, their study predates the *Brasil Sem Homofobia* initiative (Conselho Nacional de Combate à Discriminação, 2004), which played a pivotal role in raising public awareness about homosexuality and condemning hate-motivated violence. Consequently, the academic literature—particularly regarding adolescence—remains limited in its engagement with this topic.

The notions of homosexuality as both a medical condition and a choice originated in the nineteenth century. Later, the psychological perspective suggested that traumatic experiences could influence sexual orientation. While these traditional conceptions challenged stigmatizing ideas of homosexuality as a sin, they have persisted in many societies. In Brazil, homosexuality is still viewed as a medical condition (Macedo & Sivori, 2018) or a consequence of choice, deviation from religious norms or traumatic experiences (Lacerda et al., 2002). Similar patterns have been documented in other societies (Elliott et al., 2022), reflecting efforts to hold individuals accountable for their sexual orientation, which leads to criminalization and stigma.

Several studies emphasize that the religious beliefs of Brazilian teachers could influence the formation of homophobic conceptions among their students (e.g., de Jesus et al., 2024; Sartori & Miranda, 2023). Conversely, Gaspodini and Falcke (2018) suggest that family or sexual abuse experienced during childhood may also contribute to the emergence of homosexuality. Another Brazilian study claims that LGBTQ+ families could negatively impact children’s development (Vezzosi et al., 2019). These conceptions, which persist in current research, may be interpreted as justifying homophobia.

The preceding overview of traditional conceptions of homosexuality has highlighted the changing social explanatory frameworks, and underscores the importance of challenging stereotypes and prejudices, as well as fostering greater appreciation of the true meaning of sexual diversity. In the specific context of Brazil, which is characterized by the rise of more conservative social perspectives and exacerbated political tensions (Salgado, 2023), it is especially essential to address these entrenched beliefs, support young LGBTQ+ people and promote inclusive education environments (Felten et al., 2024).

### Social Stereotypes and the Complexity of the Gay Male Identity

Stereotypes are static labels that simplify social complexity and shape cultural expectations (Gonta et al., 2017) regarding specific social categories. In simple terms, homophobic stereotypes can be negative if they are based on distorted elements and they generate negative feelings; positive if they are also based on distorted elements, but they generate positive feelings; or neutral if their intention is not clear (Angulo-Brunet et al., 2024).

Social stereotypes about gay men have been varied and persistent across different cultures and historical periods. Several characteristics are often attributed to gay men. These include particular gestures, ways of speaking, clothing and even particular cultural interests. The most frequent stereotypes associated with gay men portray them as effeminate, emotional, sensitive and more inclined toward the feminine (e.g., Angulo-Brunet et al., 2024; Eguchi, 2011; Martins-Silva et al., 2012). As Herek (2015) argues, homophobia functions as a mechanism for enforcing hegemonic masculinity (Connell & Messerschmidt, 2005). By sanctioning deviations from this norm, men are compelled to reject behaviors perceived as “feminine,” thereby reinforcing a system that excludes homosexuals (Carrigan et al., 1985).

In Brazil, these stereotypes heighten the pressure on adolescents to conform to hegemonic masculinity, further marginalizing those who do not fit this standard (Matavelli, 2024). Boys exhibiting traits associated with femininity are particularly vulnerable to violence, as they are perceived as weak and subordinate (Sartori & Miranda, 2023). The concept of homohysteria (Anderson, 2011) has been introduced in the scholarly literature to describe the fear among men of being perceived as homosexual when exhibiting behaviors deemed feminized. This phenomenon serves to reinforce the rejection of, and even violence toward, individuals who deviate from dominant gender norms, irrespective of their actual sexual orientation.

One particular stereotype challenges the hegemonic representations of imposed masculinity: that of the homosexual man whose attitudes, behaviors, interests and tastes bear similarities to those of heterosexual men (Clarkson, 2005; Hunt et al., 2020). This stereotype, which provokes discomfort among homophobic individuals (Galdi et al., 2023), is linked to homonormativity (Duggan, 2002), whereby gay men adopt socially acceptable heteronormative roles to assimilate into their environment, eliciting an ambivalent response in the social imagination.

In certain contexts in Brazil, hegemonic masculinity among adolescents is shaped by norms rooted in violence, homophobia and misogyny (Matavelli, 2024). Within these settings, homosexuality is tolerated only when expressed discreetly and does not disrupt traditional masculine ideals—such as emotional restraint, physical toughness, dominance and compulsory heterosexuality. These normative frameworks serve to reinforce rigid gender roles and marginalize individuals who deviate from them (Milani & Lazar, 2017).

Other stereotypes include the idea of the manipulative, wicked gay, which has been associated with psychopaths, rapists and murderers (Sánchez-Soriano, 2023). Another stereotype is that of gays who are sick, tormented and unhappy because of their sexual orientation, often linking gay men with a life of tragedy and punishment (McLaughlin & Rodríguez, 2017). There is also the stereotype of the perverted gay, who is characterized by promiscuity, compulsive infidelity and the desire to turn heterosexual adults and even children into homosexuals too, sometimes by means of sexual aggression (Lacerda et al., 2002). This stereotype also portrays gays as carriers of sexually transmitted diseases and as habitual drug users (Anderson, 2011).

Another common stereotype is the notion that gay men possess seemingly positive characteristics, such as heightened sensitivity, a good sense of humor, physical attractiveness, wealth and an eye for fashion and art (Angulo-Brunet et al., 2024; Eguchi, 2011). However, these stereotypes, although seemingly positive, can also be extremely stigmatizing. Furthermore, certain stereotypes, such as that of gay men expressing their emotions more, could be deemed a weakness. Moreover, the idea that they are kinder, more polite or more caring (Anderson, 2011), which are positive qualities of most deconstructed masculinities (American Psychological Association [APA], 2018), can paradoxically make them more susceptible to bullying at school (Plummer, 2001).

These positive stereotypes are examples of ambivalent sexism (Glick & Fiske, 1996), as the attribution of seemingly favorable traits to gay males that, nevertheless, reinforce their subordination and maintain gender hierarchies by justifying conventional gender roles and existing power imbalances.

Nardi and Costa (2015) argue that in Brazil, prejudice against homosexuals is sometimes disguised as praise for traits like sensitivity, refinement and delicacy, and is often linked to ambivalent sexism. Gay men who fit these stereotypes may be tolerated, but only if they do not challenge the traditional male role. This means that discrimination still exists—it is just expressed in a more subtle and socially acceptable way.

In short, stereotypes about gay men have served as simplified markers of social identity. They are entrenched in the social mind-set, generating tensions and complex expectations regarding the gay community. Stereotypes also act as catalysts for the normalization of homophobic language in the education context, creating a toxic atmosphere that can lead to the stigmatization of gay boys in adolescence (e.g., Baams & Kaufman, 2023; de Carvalho & dos Reis, 2015; Elipe et al., 2022). This approach provides insight into how hegemonic masculinity shapes local dynamics in Brazil, reinforcing conceptions and stereotypes about gay men while sustaining a rigid gender hierarchy that marginalizes them (Matavelli, 2024; Milani & Lazar, 2017).

### Study Objectives

As introduced earlier, there is a gap in the scientific literature regarding Brazilian adolescents' conceptions of homosexuality. Addressing this gap is particularly urgent given the surge in conservatism in the last decade, which has impacted social and cultural dynamics in Brazil, and may have shaped these conceptions. Additionally, the lack of analysis of adolescents' expressions when referring to homosexual individuals limits our understanding of how specific content about homosexuality is manifested and perpetuated. This study aims to address this research gap by pursuing three specific objectives (OB):

- OB1: Determine adolescents’ conceptions of male homosexuality.
- OB2: Identify adolescents’ social stereotypes about gay men.
- OB3: Analyze the frequency and distribution of conceptions and stereotypes, identifying patterns and gender-based differences.

## Method

### Participants

This research used the focus group (FG) technique, which is a valuable tool for examining the everyday experiences, relationships and identities of adolescents (Bagnoli & Clark, 2010). FGs also serve as a vital means to address and mitigate any power imbalances that might exist between researchers and participants, fostering a more equitable and collaborative research environment (Adler et al., 2019). The study uses audiovisual narratives as stimuli to encourage the participants to voice their opinions and reveal their conceptions. Audiovisual narratives are a suitable tool for studying prejudices toward certain groups (Banas et al., 2020), such as LGBTQ+ people (McGuire et al., 2023). The study was designed to use narratives to help the participants to naturally and spontaneously respond to the portrayals that they were shown, ensuring good quality observations. It included three types of FGs, as will be detailed in the procedures section. The participants in each of the FGs watched two out of four narratives that were randomly assigned to them. These narratives portrayed the same protagonist in a combination of normative/non-normative male gender expressions and being identified as homosexual/heterosexual. After watching each video, the participants answered a series of questions.

A total of 178 teenagers (50% girls; age range 11–16 [*M* = 13.7, *SD* = 1.6]) participated in 45 FGs conducted across five schools in São Paulo, Brazil, with four participants per group. Among them, only three individuals self-identified as non-heterosexual.

### Measures 

#### Focus Group Script

We followed a semi-structured script designed to meet the three specific objectives of the research and which are set out in five blocks. All participants were exposed to the blocks, regardless of the school they attended:

- *Block 1* (O2): Stimulating the expression of the adolescents’ overall opinions about the characters. We presented two characters that embody stereotypical behaviors linked to male homosexuality: One challenged traditional masculinity by breaking gender norms, while the other followed them, only revealing their homosexual or heterosexual identity at the end of the narrative. The participants were asked about their impressions and descriptions of the characters’ traits, personalities and clothing, without viewing the videos.
- *Block 2* (O1/O2): Aiming to reveal the teenagers’ opinions about the stereotypes associated with homosexual people in roles that are traditionally associated with masculinity (e.g., playing football). Questions were posed to gather opinions, whether positive or negative, about challenging situations. Participants were asked why they thought the character faced homophobic harassment, such as mockery or sarcasm. For instance: “What do you think about the character’s situation with his friends? Do you believe there are reasons why they avoid playing football (or going to the bathroom) with him?".
- *Block 3* (O1/O2): Seeking to identify the participants’ stereotypes associated with male homosexuality by asking questions that explored the reasons why the victims suffered homophobic harassment. These questions aimed to generate debate about the transgression of male gender roles, the violation of mandatory homosocialization (having more female friends than male ones) and the stigma attached to male homosexuality. These questions included: Why do the others think the character is gay? Do you think the character is gay? Why?
- *Block 4* (O1/O2): Intended to identify the teenagers’ attitudes toward the contradictions that are present in preconceived ideas and to mitigate generalizations that feed prejudice. This happened when the characters revealed their sexual orientation, be that heterosexual or homosexual. The questions included: What do you think about the character being gay (or straight)? Why did you think the character was gay?
- *Block 5* (O1/O2): Focused on assessing the adolescents’ knowledge about traditional conceptions of male homosexuality and social stereotypes about gay men. The questions included: What do you know about gay men? Why do you think someone might be gay?

#### Stimuli

Four videos were created in cartoon format from two different stories that were shown at different times during the FGs (Vicente-Cruz, 2023). Each story featured a character presenting either non-normative (Story 1) or normative gender roles (Story 2). There were two different endings to each story: one where the main character identifies as gay and another where the main character identifies as heterosexual.

Story 1 featured a fairly good soccer player who was consistently picked last and subjected to homophobic remarks like “football is for men.” Story 2 was about a popular skateboarder who gets bullied for being friends with a student labeled as gay. Despite his popularity, he is ostracized by his classmates and does not understand why they reject him. In the two versions of these narratives, the main character was again revealed to be either heterosexual or homosexual.

In the videos used, two variables that shape stereotypes about male homosexuality were manipulated: gender expression and sexual orientation. The gender expression variable presented two different conditions: non-normative gender expression, which challenges the standards, structures and symbols of hegemonic masculinity ideology; and normative gender expression, which conforms to the codes linked to hegemonic masculinity. The sexual orientation variable included two conditions: homosexuality, defined as sexual attraction to individuals of the same sex and/or gender; and heterosexuality, defined as sexual attraction to individuals of the opposite sex and/or gender.

These scenarios, grounded in scientific evidence, identify sports environments and restrooms as high-risk spaces for gay boys (Plummer, 2006). This reinforces homophobia, linked to norms that privilege hegemonic masculinity (Connell & Messerschmidt, 2005) and penalize homosexuality (Herek, 2015). In sports, the pressure to conform to these standards punishes any deviation from the expected aggression and competitiveness (Anderson, 2011). Thus, these scenarios provide a framework for analyzing how traditional conceptions and stereotypes about homosexuality are activated. Based on the combination of these two variables, four types of characters and narrative scenarios were created (see Table 1)***.***

**Table 1:** Design for the 45 focus groups

Group	Presented stimulus	Videos
FG1	Non-normative homosexual + normative heterosexual	Video 1 + Video 4
FG2	Non-normative heterosexual + normative homosexual	Video 2 + Video 3
FG3	Non-normative heterosexual + normative heterosexual	Video 2 + Video 4

15 in each focal group type

*FG* Focus group

### Procedure

Students from five schools in the municipality of Sao Paulo participated in this study. Before conducting the research, authorization was obtained from the ethical committee (PUCRS Research System [SIPESQ Code: 7102] and Brazil Platform CAAE [54415816.2.0000.5336]) as well as signed consent from the legal guardians of the participating teenagers.

To validate the effectiveness of the narratives and semi-structured script, a pilot study was conducted with three focus groups (FGs). This pilot study confirmed that the materials generated the expected responses and were appropriate for the research. The FG was conducted following Krueger and Morgan’s (2014) recommendations, which suggest that a smaller group size of four participants fosters greater participation.

First, the two characters were presented on a TV screen as an introduction to the debate. As described earlier, the adolescents then watched two of the audiovisual narratives produced for the study, each representing one of the two different combinations of the main characters’ gender expression and sexual orientation (Table 1). The participants were randomly assigned to the different FGs while maintaining balance between genders in each group, with a total of four participants in each focus group.

### Data Analysis

We transcribed the 45 FGs verbatim and an external researcher reviewed them to ensure that the texts were accurate. During this process, the document was anonymized and measures were implemented to safeguard the participants’ confidentiality. The focus of the analysis was not to examine their interactions within the group, but rather to describe and identify these historical conceptions and social stereotypes. Two coders then agreed on preliminary themes and subthemes, following the phases of thematic analysis (Braun & Clarke, 2023). ATLAS.ti was used to code the data from nine FGs to refine the themes and subthemes and assess consensus. This led to a coding system consisting of two thematic blocks, five themes and 23 subthemes.

Once the subthemes were defined, all 45 focus groups (FGs) were fully coded, identifying all statements related to conceptions and stereotypes associated with homosexuality. The frequency of these statements was analyzed to quantify the presence of each themes, distinguishing between traditional conceptions of homosexuality and stereotypes about gay men, categorized as negative, positive or those that partially challenge well-established conceptions and stereotypes. This study presents the frequencies of the emerging thematic categories, both overall and broken down by gender. Although primarily a qualitative study, this supplementary procedure enables us to identify patterns and quantify the prominence of specific themes, offering a clearer perspective on the data and complementing the qualitative insights with a more tangible understanding of the findings.

## Results and Discussion

The first objective (O1) of the study is to examine how adolescents conceptualize male homosexuality, with particular attention to whether traditional understandings shape their current perspectives. The second objective (O2) is to identify the social stereotypes adolescents associate with gay men, focusing on the predominant preconceived notions and generalizations within this population. This analysis seeks to understand how such conceptions and stereotypes may influence adolescents’ attitudes and behaviors toward gay men.

Table 2 summarizes conceptions of homosexuality and social stereotypes about gay men identified among the participating teenagers. The analysis involved grouping their perceptions into two main themes: conformity with traditional conceptions and social stereotypes and tension affecting conceptions and stereotypes. Conformity involves normalizing common views of male homosexuality, accepting negative stereotypes about gay men and promoting positive stereotypes of the gay community. Tension implies the questioning of preconceived ideas about male homosexuality and doubting or rejecting the negative social stereotypes associated with gay men.

**Table 2:** Traditional conceptions of male homosexuality and social stereotypes associated with gay men among adolescents

Conformity with traditional conceptions and social stereotypes	Tension with traditional conceptions and social stereotypes
Normalization of traditional conceptions of male homosexuality	Questioning of traditional conceptions of male homosexuality
Is voluntary	Is innate
Is a sin	Is not a sin
Is a pathology	Is not a pathology
Is contagious	Is not contagious
Normalization of negative social stereotypes associated with gays	Questioning of negative social stereotypes associated with gays
Are not real men	Not all gays are effeminate
Are effeminate and/or want to be women	Not all gays can be identified
Can be identified	
Have more friendships with girls	
Do not share hobbies and tastes with heterosexuals	Can share friendships, hobbies and tastes with heterosexuals
Sexually harass heterosexuals	Do not sexually harass heterosexuals
Normalization of positive social stereotypes associated with gays	
Are happier	
Are more fun	
Are more sincere	
Are better friends	
Are more empathetic	

**Fig. 1:**
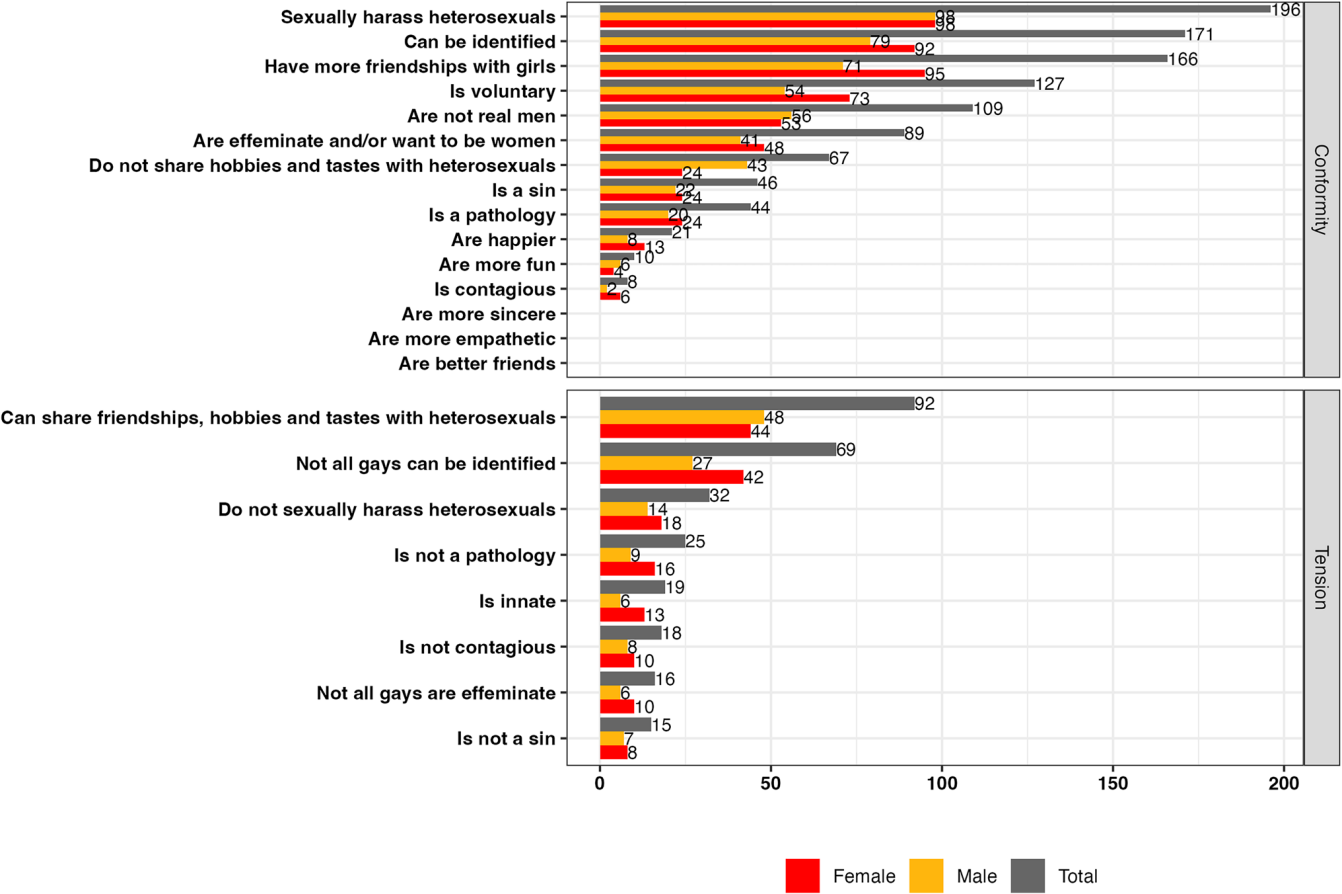
Frequencies of traditional conceptions of male homosexuality and social stereotypes associated with gay men among adolescents

Figure 1 displays the frequencies of conformity and tension with traditional conceptions and social stereotypes that emerged in the focus groups. It is important to note that some categories were identified multiple times by a single participant. The figure shows conformity with traditional conceptions and social stereotypes at the top, and tension with them at the bottom. Within each box, the data are arranged from highest to lowest based on the total count.

As shown, in terms of conformity, the most frequently identified categories are “sexually harass heterosexuals,” “can be identified,” “have more friendships with girls,” “being a voluntary choice” and “are not real men.” Conversely, in terms of tension, the most frequently identified categories are “share friendships, hobbies and tastes with heterosexuals" and "not all gays can be identified.”

When analyzing the data by participant gender, no significant differences were found across most categories. Notably, however, girls emphasized having more female friends than male friends to a greater extent than boys did, and they also perceived it as a more voluntary choice compared to boys.

### Normalization of Traditional Conceptions of Male Homosexuality and Negative Social Stereotypes Associated with Gays

Regarding traditional conceptions of homosexuality, this study underscores their persistence, such as the belief that homosexuality is a choice, a sin or a pathology (Gaspodini & Falcke, 2018; Lacerda et al., 2002; Macedo & Sivori, 2018; Vezzosi et al., 2019). This is evident in the rejection of victimized characters in the audiovisual narratives. The teenagers emphasize that the gay label entails a feeling of humiliation for the victim.

These adolescents tend to naturalize heterosexuality as a default norm, seldom considering the possibility that characters—particularly those who deviate from conventional gender norms—might be heterosexual. This pattern reflects the extent to which heterosexuality functions as the dominant frame of reference and the most socially accepted orientation among adolescents, perceived less as a personal choice and more as a default or expected condition. (“I have nothing against the character, but he seems weird to me. If I’m walking down the street and see two guys kissing, I don’t like it. If I see a man and a woman, everyone feels comfortable, they don’t even notice, but when it is a man with a man, everyone’s going to want to criticize, they’ll talk” [Boy, 13, FG3]).

The deeply entrenched notion of homosexuality as a sin has largely permeated Brazilian society, rooted in religious influences. This idea is exemplified by responses such as: “God made man to be with woman and woman to be with man. A lot of people judge, they say that anyone homosexual will go to hell” (Girl, 12, FG2). The persistence of these conceptions reinforces stigma and legitimizes homophobic discourse, attitudes and behaviors, extending beyond the educational community. In the current political context of Brazil, with its strong religious influences, this view deepens the exclusion of LGBTQ+ people and hinders progress toward greater inclusion and respect for diversity.

The idea that homosexuality is a pathology reflects the influence of past medical perspectives that classed it as a disease (e.g., “I wouldn’t hang out with him because of his attitudes. I think some people are influenced. As he doesn’t do things right [be homosexual] and might be unhealthy, I wouldn’t go out with him” [Girl, 14, FG1]). All of this supports the need to train teachers on relevant aspects of sexual diversity, which is crucial for addressing these stigmatizing views (Barreiros & Brêtas, 2024; De Jesus et al., 2024).

As observed, most traditional conceptions seem to have been normalized within a subset of Brazilian adolescents. However, no beliefs were identified in the sample suggesting that sexual orientation can be attributed to childhood experiences or traumatic family settings, as several authors have claimed (Gaspodini & Falcke, 2018; Lacerda et al., 2002; Vezzosi et al., 2019). These findings suggest the existence of a possible positive social shift among adolescents, whereby fewer prejudices are becoming ingrained in Brazilian society.

One of the most frequently identified negative stereotypes is the idea that gay men sexually harass heterosexual men. This stereotype is reinforced by the belief that gay men do not share the interests of their heterosexual peers, which justifies their exclusion from spaces such as men's restrooms and sports, where physical proximity occurs. One participant said: “I was confused because queers don’t like playing football. If the ball hits him, he goes (in a high-pitched voice): ‘Ouch, you idiot, you’re being nasty’. And if you kick the ball at him, he goes: ‘Ooh!’ Now, a guy, a faggot who’s good at football, I’ve never seen that.” (Girl, 13, FG3).

These findings suggest that when an outgroup, such as homosexuals, is perceived as highly similar and potentially threatening to the identity of the ingroup, in this case heterosexuals, the ingroup may attempt to restore the distinctiveness of their own group (Galdi et al., 2023). This idea could widen the divide between the two groups and promote discrimination. Some participants think that anyone who establishes friendships with homosexuals must also be homosexual. This situation shows how stigma and prejudice impact interpersonal relationships, hindering coexistence. For instance, one participant says: “Those who aren’t gay won’t go near them because people will say they’re gay and are trying to get off with them” (Girl, 13, FG3). These stereotypes reinforce discriminatory narratives that fuel isolation, social exclusion and violence against the LGBTQ+ community (Vicente-Cruz et al., 2025). Such attitudes may stem from homohysteria, a phenomenon that drives many adolescents to avoid associations with feminization or homosexual individuals, fearing they will be similarly labeled (Anderson, 2011).

Other common negative stereotypes include the belief that “gays can be identified” (Elliott et al., 2022; Lacerda et al., 2002), which reflects a tendency to look for and detect traits that confirm someone’s sexual orientation, but which can lead to misjudgments, as shown in this comment about the non-normative character: “My impression of him the first time I saw him was that he looked gay.” (Boy, 12, FG1).

Some participants also think that “gays have more friendships with girls,” which reinforces stereotypes of gender-segregated friendships (Plummer, 2001) or the idea that homosexual men cannot befriend heterosexual men (e.g., “I find that girls get on better with that guy, so boys are even more prejudiced because if you hang out more with girls, you’re homosexual” [Boy, 16, FG1]). Previous research has shown that this type of perception generates division and fosters homophobic discrimination (Clarkson, 2005).

In addition to these more common stereotypes, other preconceived notions, whether negative or positive, also appear in the language of adolescents. Some participants defend the idea that “gays are not real men,” which is associated with traditional notions of masculinity, where homosexuality is perceived as a lack of virility or bravery (Matavelli, 2024; Milani & Lazar, 2017). One participant said: “I think they’d tell him that if he wants to play for the football team, he’d better start dressing like a man” (Girl, 12, FG1). Furthermore, the belief among some other participants that “gays are effeminate and/or want to be women” reinforces gender stereotypes (Eguchi, 2011; Sartori & Miranda, 2023), limiting masculine expression and suggesting that homosexuality is a deviation from maleness. One participant illustrates this by saying: “There’s a boy at school who looks like a girl. The way he walks, the way he dresses, he looks like a girl. And his hair is long, he ties it in a pony tail […] He has that delicate air” (Girl, 15, FG1).

On the other hand, there were some positive perceptions about homosexuality, reflected in positive stereotypes attributed to gay men, such as being more empathetic, fun or good friends. However, these conceptions can be stigmatizing as they reinforce ambivalent sexism and create a narrowly defined model of acceptable homosexuality (Nardi & Costa, 2015). One participant’s response illustrates this belief when she says: “There are some gay people I know who are happier than others. Because they know how to have fun and they don’t care what other people say” (Girl, 11, FG2). Another reflects the belief that gay men are sincere and empathetic: “It's because a gay man tries to see both the woman's side and the man's side. So, if one day a woman goes to a gay man and asks, ‘What would you do in this situation?’ At that moment, he will try to take the woman's side and also the man's side” (Girl, 13, FG3).

These stereotypes could bolster the preconceived idea that homosexuality is associated with positive characteristics, which in turn could contribute to the idealization or stereotyping of homosexual identity. They are negative because, for young gay individuals, they dictate ways of living and expressing their identities, limiting their freedom. Such stereotypes become normalized, despite being based on distortions that shape the expression of gay individuals (Anderson, 2011). Furthermore, they set expectations that, if not met, can lead to greater rejection of gay people who have other styles of self-presentation.

### Questioning of Traditional Conceptions of Male Homosexuality and Negative Social Stereotypes Associated with Gays

In the Brazilian context, research on male homosexuality has focused on understanding negative conceptions and stereotypes (e.g., Matavelli, 2024; Sartori & Miranda, 2023; Vezzosi et al., 2019). This emphasis is justified by Brazil's leading position in global homicide rates against LGBTQ+ individuals (Observatório de Mortes e Violências LGBTI+ no Brasil, 2024), which has prompted academic focus on structural violences and their effects. This study broadens the scope by addressing not only negative conceptions and stereotypes but also the emerging changes driven by social endeavors and achievements. Although stigmatizing discourses persist and hegemonic masculinity remains the dominant framework during adolescence, the findings also indicate a growing openness to a more inclusive view of sexual diversity, showing the simultaneity of persistence and change in societal views.

The analysis reveals a potential generational shift in attitudes among certain Brazilian adolescents, who challenge traditional beliefs that have historically stigmatized homosexuality. Specifically, it highlights a tension within a subset of adolescents, with some participants actively rejecting the view that homosexuality is a voluntary choice, a belief that has long contributed to its stigmatization. These conceptions reflect an understanding of sexual orientation as an intrinsic characteristic of people, setting aside the moralistic and pathologizing views (Lacerda et al., 2002) that have pervaded the conception of homosexuality in Brazil (e.g., De Jesus et al., 2024; Gaspodini & Falcke, 2018; Martins-Silva et al., 2012; Milani & Lazar, 2017).

These results partly challenge previous studies which commonly report that homophobia is extremely common among Brazilian adolescents (de Carvalho & Reis, 2015; Martins-Silva et al., 2012) without taking into consideration the shifting societal perceptions that include more positive views of same sex people and relationships. Questioning and rejecting stigmatizing conceptions, such as the idea that homosexuality is a sin, a disease or something contagious, highlights a groundswell leading to greater inclusion of sexual and gender diversity. This perspective, which involves the rejection of psychologizing conceptions, and integrates homosexuality into social normality, is crucial for reducing stigma. Moreover, it promotes a more inclusive coexistence, which can have beneficial effects on both the social and psychological well-being of LGBT+ individuals.

Among the most relevant contributions of the study is the lack of resistance toward the positive stereotypes associated with gay men among some individuals in the sample. There may be diverse reasons why these stereotypes are not questioned. They might be due to the internalization and general acceptance of these stereotypes in a society where they are perceived as a normal and accepted part of gay identity. There is evidence of the internalization of society's positive biases toward stereotypically masculine traits among gay men (Hunt et al., 2020).

As a result, academic attention has predominantly centered on studying these structural violences and their impacts. However, this study expands the scope by exploring not only negative conceptions and stereotypes but also the emerging changes fueled by social movements and achievements. While stigmatizing discourses persist, the findings suggest an openness toward a more inclusive view of sexual diversity, which may contribute to reducing stigma and discrimination.

However, it is essential for adolescents to be made aware of the persistence of both negative and positive stereotypes. Teaching them to critically engage with these stereotypes is necessary, as without exposure to alternative or counter-narratives, they are likely to accept them uncritically. Indeed, the absence of discussions about the diversity within gay identity may contribute to the normalization of such stereotypes, further reinforcing their unchallenged status.

It is possible that these ideas are influenced by the feminist and LGBTQ+ movements in their pursuit of equal rights, which has become a global phenomenon. As previous studies suggest (Morrison & Bearden, 2007), the results indicate that girls are more likely than boys to endorse positive conceptions of gay men.

### Implications of the Study

This study confirms the ongoing presence of negative conceptions and stereotypes surrounding male homosexuality during adolescence. These findings are consistent with previous research that has highlighted stigmatization in educational contexts (McGuire et al., 2023), particularly in institutions lacking active inclusive policies (Sartori & Miranda, 2023; Souza et al., 2017; Vicente da Cruz et al., 2021). The frequent use of stigmatizing expressions illustrates how conservative discourses continue to shape youth identity in Brazil (Gaspodini & Falcke, 2018; Macedo & Sivori, 2018).

Given this reality, it is crucial to strengthen teacher training on sexual diversity to address discourses that perpetuate invisibility and exclusion (Barreiros & Brêtas, 2024; De Jesus et al., 2024). While schools remain spaces where cisheteronormative discourses are reinforced, they also have the potential to transform if strategies are developed to challenge negative views of homosexuality, fostering an inclusive educational environment (Felten et al., 2024). This requires making LGBTQ+ realities visible in the curriculum and promoting school policies that counteract these harmful conceptions. Additionally, it is essential to provide teachers and students with the tools to confront and mitigate school violence (Vicente-Cruz et al., 2025).

Furthermore, the study’s results highlight that hegemonic masculinity remains the dominant framework during adolescence in terms of defining what is considered acceptable within male identity (Connell & Messerschmidt, 2005), despite emerging shifts toward more inclusive views. The reliance on hegemonic masculinity suggests that young people continue to reinforce rigid gender norms, punishing any deviation that challenges normative masculinity (Herek, 2015). This finding is particularly significant in the Brazilian context, where homophobia is deeply intertwined with the construction of virility and male competitiveness (Matavelli, 2024). The exclusion of homosexual adolescents from sports and school environments reflects the persistence of a masculinity model that equates homosexuality with weakness and vulnerability (Milani & Lazar, 2017). In response, public educational policies should prioritize initiatives that promote gender equity and recognize sexual diversity in educational settings. Moreover, there is an urgent need to develop promote more inclusive and egalitarian models of masculinity that not only acknowledge sexual diversity but also challenge traditional ideals of virility that foster discrimination and social exclusion (American Psychological Association, 2018). To address these stigmatizing conceptions and stereotypes, it is essential to implement intervention programs targeting gender socialization. These programs should focus on deconstructing stereotypes from early childhood, creating more inclusive spaces and ensuring the representation of positive LGBTQ+ role models in media and education (Angulo-Brunet et al., 2024; Sánchez-Soriano et al., 2023; Soto-Sanfiel et al., 2025). By adopting these strategies, we can reduce stigma and foster more inclusive societies that embrace sexual and gender diversity.

### Conclusions

This study reveals the persistence, within a subset of Brazilian adolescents, of deep-rooted traditional conceptions, social stereotypes and prejudice toward gay people and the stigmatization of male homosexuality. The findings highlight the relationship between negative stereotypes and traditional gender representations, which increases the discrimination and isolation of homosexual people. All of this underscores the importance of fostering attitudinal changes in formal and informal settings to promote respect, inclusion and equality. In addition to advancing academic knowledge, the results of this study could help educators and institutions identify and address conceptions about gay individuals within school settings.

The study reveals that one of the most persistent stereotypes among the participants is the entrenched perception of homosexuality as a sin, which is undoubtedly influenced by religious beliefs and is deeply ingrained in Brazilian society. In this regard, it is paramount to consider that recent research (de Jesus et al., 2024; Sartori & Miranda, 2023; Vicente da Cruz et al., 2021) highlights that educators' religious convictions fuel homophobia among students. This helps to underscore a concerning aspect of the Brazilian education panorama: Teachers not only overlook diversity and various forms of prejudice, but there are shortcomings in their training which are exacerbated by religious fundamentalism, hindering sociocultural approaches to sexual education. Consequently, the results of this research reveal the need to incorporate teacher training policies in relevant aspects of sexual diversity to counteract the entrenched stigmas. Together with the deconstruction of Brazilian adolescents’ emphasis on perceiving gay men through stereotypes, the tendency of some Brazilian teachers to conceal prejudiced behaviors needs to be neutralized. For instance, Barreiros and Brêta (2024) evidenced that despite Brazilian students openly admitting the existence of gays and homophobia, teachers either refute or ignore the verbal violence perpetrated by students, attempting to deny the existence of homophobia or homosexuals in schools.

The results indicate differences in conceptions of homosexuality. However, there is still a lack of resistance against stereotypes that might seem positive, but can also be stigmatizing (Angulo-Brunet et al., 2024; Morrison & Bearden, 2007). This could be related to a lack of exposure to critical social discourse on diversity in gay identity. However, some adolescents in the sample explicitly reject stereotypical and discriminatory ideas conveyed through homophobic language expressions, deeming them unjust. This is especially noteworthy given that adolescents are at a formative stage of their lives, where peer influence significantly shapes social perceptions.

The findings of this study reveal the persistence of historical views on male homosexuality among Brazilian adolescents, underscoring the need for further research into how these discourses intersect with contemporary representations of masculinity. Additionally, future research could delve into how these discourses are interpreted and negotiated in different educational contexts, enriching our understanding of sexual identity development during adolescence.

Nonetheless, societies should not burden their youth alone with the responsibility of combating discrimination against LGBTQ+ individuals. Consequently, future research could investigate effective education strategies to address and challenge entrenched stereotypes and prejudice with regard to homosexuality among adolescents; examining how media and cultural portrayals of gay identity influence the formation of stereotypes and attitudes toward gays among young people. Knowledge also needs to be furthered on how the internalization of stereotypes can affect the mental health and well-being of young LGBTQ+ people in particular settings. Finally, there is a need to develop specific programs aimed at reducing stigma and prejudice toward LGBTQ+ individuals in education contexts. Research of these topics could generate a more complete understanding of how stereotypes and perceptions of gender and sexual identities impact society, and also contribute to the design of more effective strategies to promote inclusive education in adolescent settings.

### Limitations

This study, through exploratory qualitative research, has identified the views of Brazilian adolescents on homosexuality. As one of the most populated countries in the Global South and a global leader in violence against LGBTQ+ individuals, Brazil provides a critical context for understanding these perceptions. The findings serve as a valuable starting point for discussions on the diverse realities across this region. However, future studies in other cultural contexts, both within the Global South and beyond, with varying dominant religions, are needed to evaluate the broader applicability of these results. Given its design and scope, this research does not claim to establish a cause-and-effect relationship between participants' beliefs and behaviors, recognizing that both may be influenced by factors such as social desirability, peer pressure or personal safety. In real-world contexts, individuals may behave differently from their stated beliefs, particularly on sensitive topics like homosexuality. Some interview questions were formatted as yes/no items, which may have biased responses toward affirmation, thereby limiting the depth of participant input. Furthermore, a double-barreled question, which included an "or," may have caused confusion, potentially compromising the clarity of the data. Future research should prioritize the use of open-ended questions that are clearly framed to elicit more nuanced and reliable responses. Thus, future research should explore this relationship using methodologies that account for these factors while ensuring the well-being of participants. In the meantime, the findings offer important insights into the Brazilian educational context.

## Data Availability

Due to the nature of the research and to ethical issues, supporting data are not available.
